# Influence of Lateral Hard‐Tissue Grafting on Peri‐Implant Health or Disease: A Cross‐Sectional Study

**DOI:** 10.1111/cid.13414

**Published:** 2024-11-09

**Authors:** Amira Begić, Frank Schwarz, Karina Obreja, Iulia Dahmer, Julie Recktenwald, Puria Parvini, Ausra Ramanauskaite

**Affiliations:** ^1^ Department of Oral Surgery and Implantology Goethe University Frankfurt Frankfurt Germany; ^2^ Faculty of Medicine, Institute of Biostatistics and Mathematical Modelling Goethe University Frankfurt am Main Germany

**Keywords:** alveolar ridge augmentation, dental implant, guided bone regeneration

## Abstract

**Aim:**

To investigate the influence of lateral hard‐tissue grafting performed simultaneously to implant placement on peri‐implant health or disease.

**Materials and Methods:**

A total of 299 patients exhibiting 897 implants placed either simultaneously with lateral bone grafting using a bovine bone mineral with or without adjunctive native collagen membrane (*n* = 131/269 patients/implants; LatGr group) or at pristine bone sites without lateral bone grafting (*n* = 168/628 patients/implants; NoGr group) were enrolled in this cross‐sectional analysis. Clinical outcomes (i.e., modified plaque index (mPI), bleeding on probing (BOP), probing depth (PD), mucosal recession (MR)), keratinized mucosa (KM), and the frequency of peri‐implant disease were evaluated. Univariate and multiple ordinal regression analyses with mixed effects were conducted to identify factors associated with peri‐implant disease.

**Results:**

After a mean follow‐up period of 59.47 ± 24.66 months, mPI, BOP, and KM values were significantly higher in the control group, whereas no difference between the groups was found for PD and MR. Peri‐implantitis was diagnosed in 31.30% of patients and 13.4% of implants in the LatGr group, and in 32.74% of patients and 24.8% of implants in the NoGr group. The corresponding values for peri‐implant mucositis at the patient and implant level in the LatGr and NoGr groups were 29.77% and 29.0%, and 26.19% and 28.5%, respectively. Implants of the LatGr group were associated with a significantly lower chance to be affected by peri‐implant disease (OR = 0.69, 95%‐CI: (−0.72, −0.03), *p* = 0.032). The presence of plaque at implant sites and smoking significantly correlated with peri‐implant disease (OR = 2.92, 95%‐CI: (2.11, 4.03), *p* < 0.001 and OR = 0.22; 95%‐CI: (0.06, 0.84), *p* = 0.027, respectively).

**Conclusions:**

Lateral hard‐tissue grafting performed simultaneously with implant placement demonstrated comparable peri‐implant tissue health to pristine bone sites without lateral bone grafting.

## 1 Introduction

Tooth extraction is followed by inevitable dimensional changes of the alveolar ridge, with a more pronounced volume reduction occurring in the horizontal than the vertical dimension [1, 2]. Consequently, prosthetically driven implant insertion is often accompanied by the occurrence of a dehiscence‐type defect on the facial bone wall, which requires simultaneous lateral hard‐tissue grafting [3, 4]. Lateral bone augmentation procedures are effective in treating dehiscence‐type defects, resulting in a mean defect resolution of 81% [5]. The reconstruction protocols employing a combination of a bone substitute material and a barrier membrane were found to be more effective in obtaining a defect fill compared with the use of a bone filler or a membrane alone [6, 7]. In fact, the spontaneous healing of facial peri‐implant dehiscence bone defects resulted in considerably greater vertical bone loss during the initial healing phase than at the implant sites treated with simultaneous lateral hard‐tissue augmentation, thus underlining the clinical relevance of this intervention [8]. It is worth noticing that guided bone regeneration (GBR)‐supported hard‐tissue grafting protocols are today considered well‐established and highly reliable surgical techniques for treating localized bone defects [9, 10].

One recent systematic review and meta‐analysis advocated comparable changes in bleeding on probing (BOP), probing depth (PD) values, and marginal bone‐level changes between implants placed along with or without lateral hard‐tissue grafting [11]. Likewise, a recent cross‐sectional study failed to identify any negative influence of simultaneous lateral bone augmentation employing a bone substitute and a barrier membrane on the maintenance of peri‐implant tissue health throughout a mean follow‐up period of 10 years [12]. In particular, peri‐implant mucositis and peri‐implantitis were diagnosed in 68% and 5% of patients in the test group (i.e., patients with implants placed with simultaneous lateral hard‐tissue grafting), and 61% and 10% of patients in the control group (i.e., patients with implants inserted in a native bone without a need for hard‐tissue grafting), respectively [12]. The contradicting clinical data, however, pointed toward a link between lateral bone augmentation procedures performed along with implant placement and increased peri‐implantitis risk (OR = 2) [13]. In this context, it should be acknowledged that despite the grafting procedures, the reported fill of dehiscence‐type defects following lateral bone augmentation was wide (56%–97%), thus suggesting that a significant number of dehiscence defects still persist [5]. Subsequently, as shown by the previous clinical data, implants with residual defects on a facial bone wall possess a higher risk for developing biological complications [14]. Given the existing contradiction in the current literature, the aim of the present cross‐sectional analysis was to investigate the influence of lateral hard‐tissue grafting performed simultaneously with implant placement on peri‐implant tissue health status. It is hypothesized that implants placed along with the simultaneous lateral hard‐tissue grafting show more clinical signs of inflammation and are subsequently more frequently affected by peri‐implant mucositis and peri‐implantitis comparted to the implants inserted in the native bone without a need for hard‐tissue grafting.

## 2 Material and Methods

### 2.1 Study Design

The present cross‐sectional analysis included 299 partially or fully edentulous patients (173 females and 126 males) exhibiting 897 two‐piece titanium implants. Patients were enrolled during the yearly regular maintenance visits at the Department of Oral Surgery and Implantology, Goethe University, Frankfurt, between January 2020 and December 2020. All implants were placed in the Department of Oral Surgery and Implantology, Goethe University, Frankfurt, between 1992 and 2019.

Thirty‐six patients with 109 implants (with hard‐tissue grafting: 13 patients/39 implants, without hard‐tissue grafting: 23 patients/70 implants) were included in a previous analysis but at different follow‐up time‐points [12]. Each patient received a detailed description of the procedure, and an informed consent form was obtained prior to the screening. The procedures in this study were in accordance with the Declaration of Helsinki, as revised in 2013, and the study protocol was approved by the local ethics committee (registration number: 20/545).

### 2.2 Patient Selection Criteria

The following inclusion criteria were applied for patient selection:

- Patients with > 18 years of age rehabilitated with at least one screw‐type two‐piece implant;
- Periodontally healthy patients and patients with treated chronic periodontitis enrolled in regular maintenance care;
- Nonsmokers and smokers;
- Attendance of a yearly routine implant maintenance appointment.

Patients were excluded for the following conditions: systemic diseases that could influence the outcome of the therapy, such as diabetes (HbA1c > 7), osteoporosis; a history of malignancy, radiotherapy, chemotherapy, immunodeficiency, or antiresorptive therapy; and pregnancy or lactation at the last follow‐up.

### 2.3 Treatment Protocol

Two‐piece implants were placed in a prosthetically driven position. Implants in the NoGr group were inserted in a native bone and displayed an intact vestibular alveolar bone wall without the need for a lateral bone grafting procedure. Implants in the LatGr group exhibited dehiscence‐type defects at the vestibular aspect, which were simultaneously filled with a particulated bovine bone mineral (Bio‐Oss spongiosa granules sized 0.25–1 mm, Geistlich, Wolhusen, Switzerland) with or without adjunctive application of a native collagen membrane (Bio‐Gide, Geistlich, Wolhusen, Switzerland). Submerged and transmucosal healing modus was chosen for control and test sites for a healing period of 2–4 months. The implants in both test and control groups were restored either with fixed or removable prosthetic restorations.

### 2.4 Implant and Implant‐Site Characteristics

The following study variables were assessed at both test and control implants:

(1) implant functioning time, (2) implant location (i.e., upper or lower jaw, anterior (i.e., canine to canine) or posterior (i.e., premolar and molar region) segments), (3) implant diameter and length, bone quality, and (4) type of prosthetic restoration.

### 2.5 Clinical Measurements and Case Definitions

The following clinical parameters were registered at each implant site using a conventional periodontal probe:

(1) Modified plaque index (mPI) (Löe 1967.), (2) BOP (%)—measured as presence/absence, (3) PD—measured from the mucosal margin to the probable pocket (mm), (4) suppuration (Sup)—measured as presence/absence, (5) mucosal recession (MR)—measured from the restoration margin to the mucosal margin (mm), and (6) keratinized mucosa (KM) (mm).

For clinical parameters such as mPI, BOP, PD, and MR, measurements were performed at six sites per implant: mesiobuccal (mb), midbuccal (b), distobuccal (db), mesiooral (mo), midoral (o), and distooral (do). KM measurement was performed at three aspects per implant: mesiobuccal (mb), midbuccal (b), and distobuccal (db).

Three calibrated examiners assessed all clinical measurements (AR, AB, and KO). For the calibration, repeated measurements were performed with a 10‐min interval of the assessed clinical parameters in five patients with a total of 15 implants. The calibration was acceptable when repeated measurements were similar > 95% level. The calculated mean interexaminer variability between the repeated measurements for the assessed clinical parameters ranged from 96.8% ± 3.5% to 98.5% ± 0.65%.

The presence of peri‐implant disease at each implant site was assessed according to the following criteria [15]:

Peri‐implant mucositis is defined as the presence of BOP and/or suppuration with gentle probing, and an absence of bone loss beyond crestal bone‐level changes resulting from initial bone remodeling.

Peri‐implantitis is defined as the presence of BOP and/or suppuration on gentle probing and the presence of progressive bone loss beyond crestal bone‐level changes resulting from initial bone remodeling.

### 2.6 Radiographic Assessment

Panoramic radiographs were obtained at least 12 months following the placement of the final prosthetic reconstruction (i.e., baseline) and the presence of clinical signs of inflammation (i.e., BOP/Sup) detected during the yearly maintenance visits. To evaluate the bone‐level changes at the implant sites, the obtained radiographs were compared with the baseline radiographs. After digitalization of the analog radiographs performed before 2012 (Microtek ScanMaker i800 Plus, Hsinchu, Taiwan; LaserSoft Imaging AG, Kiel, Germany), measurements (i.e., bone‐level changes between the baseline and follow‐up radiographs) were performed employing the Sidexis 4 software (Sirona Dental Systems GmbH, Bensheim, Germany). The measurement scale was based on the known implant length. Implant shoulder, the rough borderline of tissue‐level implants, was defined as a zero point. Two reference horizontal lines were used: one marked the most coronal point of the peri‐implant bone crest at mesial and distal sites (BC), and another traced the implant's most apical point (AP). Vertical lines parallel to the reference line crossing the long axis of the implant were traced perpendicularly to the BC and AP at mesial and distal sites. The higher amount of bone loss between the mesial and distal aspects was taken into account. Differences between the baseline (T0) and last radiographic measurements (T1) were compared. One previously calibrated examiner (JR) performed all measurements. The calculated mean absolute difference of the repeated measurements assessed at 10 implants was 0.10 ± 0.05 mm with an estimated intraexamined correlation coefficient (ICC) = 0.89 (95% confidence interval: (0.58, 0.97)).

### 2.7 Statistical Analysis

The statistical analysis was conducted using the software program R (R Core Team, 2021, packages “ordinal” and “performance”). For each clinical variable, descriptive statistics were conducted.

For the statistical analysis, the implant was considered as statistical unit. Prevalence of peri‐implant diseases were estimated at patient and implant levels. To account for the fact that some patients exhibited more than one implant, mixed models with the patient as random effect were considered. Univariate ordinal regressions with mixed effects were conducted for the dependent variable diagnosis (healthy/peri‐implant mucositis/peri‐implantitis) and predictors for peri‐implant disease: lateral augmentation (test/control group), jaw category (upper/lower jaw), alveolar bone quality (soft/normal/hard), implant diameter (mm), category KM (KM < 2 mm/KM ≥ 2 mm), mean MR (mm), category mPI (mPI = 0/mPI > 0), and type of suprastructure (fixed/removable). The significant predictors from the univariate regressions were then included together in a multiple ordinal regression with mixed effects considering the gender and the smoking status of the patient as a confounder. For the evaluation of the goodness‐to‐fit of the multiple regression model, a likelihood ratio test comparing the full model and the reduced model (only with confounder) is performed. The (conditional) Nakagawa's Pseudo R‐squared is computed for the full model as a further goodness‐to‐fit measure of the model. To compare the clinical parameters, that is, mPI, BOP, PD, MR, KM, between the groups, univariate linear, and logistic regressions with mixed effects were performed. Results were rated significant at *p* < 0.05%.

A sample size of 299 patients (168 in the control group and 131 in the test group), considering one implant per patient, was sufficient to detect an odds ratio of 1.83 for the diagnosis in the two groups with a power of at least 80% at a significance level of alpha = 5%. The power calculation was performed with PASS 2022.

## 3 Results

Descriptive demographic and implant‐related data are depicted in Table 1.

**TABLE 1:** Descriptive demographic and implant‐related data.

Variable	Total	LatGr group	NoGr group
No. of patients (*n*)	299	131	168
No. of implants (*n*)	897	269	628
Age (mean ± SD)	67.07 ± 12.21	65.24 ± 12	68.51 ± 11.48
Nonsmokers	291	127	164
Smokers	8	4	4
Gender (female/male) (*n*)	173 /127	74/57	99/69
Follow‐up time* (min‐max/mean ± SD) (months)	24–220/59.47 ± 24.66	24–120/55.16 ± 24.37	24–220/61.32 ± 24.56
Location of implant			
Upper/lower jaws (*n*)	366/531	121/148	245/383
Anterior**/posterior regions (*n*)	234/663	76/193	158/470
Prosthetic restoration Fixed/removable (*n*)	696/175	218/45	478/130
Reconstruction protocol:			
Bone substitute + membrane (*n*)	221	221	
Bone substitute alone (*n*)	48	48	
Implant diameter (*n* of implants)			
3.0 mm	1	0	1
3.3 mm	1	1	0
3.5 mm	749	226	523
3.75 mm	4	3	1
3.8 mm	2	2	0
4.0 mm	9	1	8
4.1 mm	11	4	7
4.3 mm	1	0	1
4.5 mm	107	29	78
5.5 mm	12	3	9
Implant length (min‐max) (mm)	5.0–17	6.0–17	5.0–17
Implant type: Ankylos/Straumann/other	597/8/22	237/2/6	360/6/16
Bone quality (soft/normal/hard) (*n*)	207/426/122	71/108/41	136/318/81

* The time > 12 months from the final prosthetic restoration to the last follow‐up.

** From canine to canine.

In total, 131 patients (mean age: 65.24 ± 12.09, 74 females, 57 males) with 269 implants (mean functioning time: 55.16 ± 24.37 months) were enrolled in the test LatGr group. The NoGr group included 168 patients (mean age: 68.51 ± 11.48, 99 females, 69 males) exhibiting 628 implants (mean functioning time 61.32 ± 24.56 months).

The majority of the implants in the LatGr and NoGr groups were located in the lower jaw (test: 121 upper jaw, 148 lower jaw, control: 245 upper jaw, 383 lower jaw). In the LatGr group, lateral hard‐tissue grafting at the majority of implants (*n* = 221) was performed using bone filler and a barrier membrane, and at the remaining 48 implants, bone filler alone was employed to fill the dehiscence‐type defects. The implants in both LatGr and NoGr groups were mainly restored with fixed prosthetics (test: 81%, control: 76%).

### 3.1 Clinical Measurements

The clinical measurements are presented in Table 2.

**TABLE 2:** Clinical parameters (mean ± SD) at the implant level.

Clinical parameters (implant level)	LatGr group (mean ± SD)	NoGr group (mean ± SD)	*p*
Modified plaque index (mPI) mPI > 0 (%)	0.22 ± 0.30 45.58	0.33 ± 0.38 58.76	0.048 < 0.001
Bleeding on probing (%)	42.38	53.34	0.042
Probing depth (mm)	2.53 ± 0.88	2.53 ± 1.00	0.75
Mucosal recession (mm)	0.25 ± 0.55	0.23 ± 0.57	0.61
Keratinized mucosa (mm)	2.83 ± 2.06	2.54 ± 1.78	0.01

Mean mPI scores and BOP values were significantly higher in the NoGr group (mPI: test: 0.22 ± 0.30; control: 0.33 ± 0.38; *p* = 0.048; BOP test: 42.38%, control: 53.34%, *p* = 0.042). The mean KM values were significantly higher in the LatGr group (2.83 ± 2.06 mm versus 2.54 ± 1.78 mm, *p* = 0.01). The mean PD (test: 2.53 ± 0.88 mm, control: 2.53 ± 1.00, *p* = 0.75) and MR values (test: 0.25 ± 2.06 mm, control: 0.23 ± 0.57 mm, *p* = 0.61) were comparable between the two groups.

### 3.2 Prevalence of Peri‐Implant Disease

The frequency distribution of peri‐implant disease in the LatGr and NoGr groups is summarized in Table 3.

**TABLE 3:** Prevalence of peri‐implant health and disease.

Diagnosis *n* (%)	Patient level NoGr	LatGr	Implant level NoGr	LatGr
Healthy	69 (41.07%)	51 (38.93%)	293 (46.7%)	155 (57.6%)
Peri‐implant mucositis	44 (26.19%)	39 (29.77%)	179 (28.5%)	78 (29.0%)
Peri‐implantitis	55 (32.74%)	41 (31.30%)	156 (24.8%)	36 (13.4%)

According to the given case definitions, 39 (29.77%) of the patients in the LatGr group and 44 (26.19%) of the patients in the NoGr group were diagnosed with peri‐implant mucositis, while peri‐implantitis was diagnosed in 41 (31.30%) of the patients in the test and in 55 (32.74%) of the patients in the NoGr group. At the implant level, the corresponding values amounted to 29.0% and 13.4% in the test group, and 28.5% and 24.4% in the NoGr group, respectively. Accordingly, peri‐implant health was recorded for 57.6% of implants in 38.93% of patients in the test group and for 46.7% of implants in 41.07% of patients in the NoGr group.

### 3.3 Regression Analyses

The results from the univariate ordinal regressions with mixed models are presented in Table 4.

**TABLE 4:** Estimates and *p* for the univariate regression analyses.

Predictor	Estimate	*p*
Lateral bone augmentation	−0.46	0.0103
Jaw category (upper/lower jaws)	0.17	0.297
Bone quality	0.20	0.159
Implant diameter (mm)	−0.19	0.36
Category KM (KM mid. buccal ≥ 2)	−0.18	0.311
Mean MR	0.18	0.226
Category mPI (mPI > 0)	1.07	< 0.001
Type of prosthesis (fixed/removable)	0.19	0.444

Based on the univariate regression with mixed models, simultaneous lateral hard‐tissue grafting was found to be associated with a significantly reduced chance of an implant being diagnosed with peri‐implant disease (estimate: −0.46, OR = 0.63; *p* = 0.0103). The presence of plaque was also found to be significantly correlated with peri‐implant disease (estimate: 1.07, OR = 2.92; *p* < 0.001).

According to the multiple ordinal regressions with mixed effects, the presence of plaque was found to be associated with an increased chance (OR = 2.92; 95%‐CI: (2.11, 4.03)) of an implant being diagnosed with peri‐implant disease (estimate: 1.07; 95%‐CI: (0.74, 1.39), *p* < 0.001). Furthermore, the lateral hard‐tissue grafting was significantly associated with a lower chance (OR = 0.69 with 95%‐CI: (0.49, 0.97)) of an implant being diagnosed with peri‐implant disease (estimate: −0.38; 95%‐CI: (−0.72, −0.03)) of being in a higher category for the disease (*p* = 0.032). Smoking habit was associated with a higher risk of an implant being diagnosed with peri‐implantitis (OR = 0.22; 95%‐CI (0.06, 0.84); estimate = −1.5, *p* = 0.027). There was no significant association between the gender of the patient and the probability of the implant of being diagnosed with peri‐implant disease.

The computed marginal pseudo R‐squared of the full model was 0.274. The full model explained the data significantly better than the reduced one (without predictors; likelihood ratio test, *p* < 0.001).

## 4 Discussion

The present cross‐sectional study aimed at evaluating the influence of lateral hard‐tissue augmentation performed simultaneous to implant placement on peri‐implant health. The analysis was based on 299 patients with 897 implants, of which 131 patients with 269 implants were referred to the LatGr group (i.e., implants placed along with the lateral hard‐tissue grafting) and 168 patients with 628 implants were assigned to the NoGr group (i.e., implants placed in a native bone).

Based on the present data analysis, peri‐implant mucositis and peri‐implantitis in the LatGr group affected 29.77% and 31.30% of the patients, and 29% and 13.4% of the implants, respectively. In the NoGr group, peri‐implant mucositis and peri‐implantitis were diagnosed in 26.19% and 32.74% of patients, and 28.5% and 24.8% of implants, respectively. Thus, the prevalence of both diseases were comparable in the LatGr and NoGr group patients, though a higher prevalence of peri‐implantitis was documented at implants in the NoGr group. The regression analysis further showed simultaneous lateral hard‐tissue grafting to be significantly associated with a lower change of an implant to be affected by peri‐implant disease (OR = 0.67, 95%‐CI: (0.47, 0.94)). The present results are consistent with those previously reported in one cross‐sectional analysis, which indicated a higher prevalence of peri‐implantitis for implants placed in native bone compared to implants inserted with simultaneous lateral hard‐tissue grafting (8% versus 5% of implants, respectively) [12]. In corroborations to the present findings, the prevalence of peri‐implant mucositis was comparable between the implants placed with or without simultaneous lateral hard tissue grafting (test: 66% of implants, control: 64% of implants) [12]. The current findings also align with the results of one former randomized clinical trial that investigate the effects of lateral bone grafting at dental implants featuring dehiscence‐type defects (≤ 5 mm) at the vestibular aspect compared to the implant sited left for a spontaneous defect healing (i.e., without grafting) [16]. The clinical assessment of implant sited after a mean follow‐up period of 7.5 years revealed comparable mean PD and sulcus bleeding index values in both groups [16]. None of the implants developed peri‐implantitis lesions. The further radiographic analysis of the implant sites revealed a significantly higher defect fill at implants treated with hard‐tissue grafting, thus justifying the efficacy of the procedure in terms of defect fill and maintenance of peri‐implant tissue health [16]. On the other hand, one retrospective study identified hard‐tissue augmentation procedures performed at the time of implant insertion to be a predictor for peri‐implantitis (PD ≥ 4 mm + BOP/Sup + bone loss ≥ 3 mm compared with the radiograph taken at the time of prosthesis delivery; odds ratio = 2.35, *p* = 0.0008) [13]. Bone augmentation procedures included both, GBR and bone expansion, without specifying the surgical protocols or biomaterials [13].

According to the current findings, significantly higher mean mPI, BOP, and KM values were detected at the implants in the NoGr group, whereas no significant difference between the groups was found for the mean PD and MR measurements. This finding contradicts the ones reported in one previous cross‐sectional study, where no significant difference was found between the implants placed with lateral hard‐tissue grafting and those placed in the native bone in terms of BOP and mPI values [12]. In fact, the mean PD measurements were even significantly higher at implants in the test group (i.e., placed with simultaneous hard‐tissue grafting) [17]. The latter finding might be at least partly related to the residual defects and subsequently exposed rough implant surfaces that persist following the lateral hard‐tissue grafting, which, as documented by the previous clinical study, increases the risk for peri‐implant disease [14].

Upon further analysis of the present data, the presence of plaque was found to be directly associated with an increased chance for peri‐implant disease. In fact, in the control group, significantly higher mPI indices were directly associated with significantly higher BOP value. The present outcome aligns with the results of the previous clinical investigations that showed poor plaque control to be the strongest statistical predictor for peri‐implantitis [18, 19, 20, 21]. Furthermore, as shown by the prospective clinical investigation, almost half of the patients with peri‐implant mucositis, who did not adhere to a regular supportive therapy, developed peri‐implantitis lesions over a 5‐years period, thus confirming that poor plaque control and lack of regular maintenance therapy directly correlate with peri‐implant disease [22].

Although clinical data identifying “smoking” as a potential risk factor/indicator for peri‐implantitis remain inconclusive, the present analysis revealed that smoking habit was associated with a significantly higher risk for peri‐implantitis. In fact, as shown by one recent analysis, smoking habit directly associated with the severity of peri‐implantitis‐related bone defects [23]. Emerging clinical evidence points to the relevance of KM in dental implants for the maintenance of peri‐implant tissue health. Specifically, a lack of or reduced amount of KT was found to be significantly correlated with higher plaque values, soft‐tissue inflammation, marginal bone loss, and higher risk for peri‐implantitis [17]. Based on the present data set, the regression analysis failed to identify KM as a risk factor for peri‐implant disease. However, when interpreting this outcome, it is important to underline that the mean KT width in both groups was > 2 mm, which may explain the lack of association between KT and peri‐implantitis tissue health or disease.

The regression analyses failed to identify any influence of implant diameter, MR, bone quality, implant location (upper/lower jaw), and type of prosthetic restoration upon the prevalence of peri‐implant disease. Contrary to this are the findings, one previous analysis reported on a significant association between a larger implant diameter (i.e., 4.5–5.5 mm vs. 3.5 mm) and increased PD values of 4–6 mm [12]. More recently, one cross‐sectional analysis identified implant diameter > 3.75 mm to be positively associated with the diagnosis of peri‐implantitis (OD = 3.638; *p* = 0.012) [24]. As speculated by the authors, a larger implant diameter may lead to an implant location being outside of the bony envelope, resulting in uncontained peri‐implant defects with a compromised regenerative potential [12]. The aforementioned association could not be confirmed by the present findings, which might at least partly be attributed to the fact that a majority of the implants in both groups had a diameter of 3.5 mm. Furthermore, contrary to the current findings, the previous clinical studies pointed to a higher risk for peri‐implant disease at implants supporting removable prosthesis compared to those restored with the fixed reconstructions and those located in the maxilla [25, 26, 27]. The later finding, as hypotheses by the authors, may be attributed at least partly to “the compact nature of the mandibular bone providing more resistance to the inflammatory infiltrate, in contrast to the trabecular nature of the maxillary bone with marrow spaces” [27]. In the present data analysis, implants were quite equally distributed in the upper and lower jaws in both groups, and most of the implants supported fixed restorations, which might explain the lack of an association between the prosthesis design and peri‐implant tissue disease.

When interpreting the findings of the present analysis, one should keep in mind a considerably lower number of implants included in the LatGr group compared to the NoGr group. Furthermore, a majority of the implants were located in posterior regions and supported fixed prosthetic restoration, which in turn might have contributed to the outcomes of the analysis. In addition, the definition of peri‐implantitis in the present analysis was based on the presence of marginal bone‐level changes along with the presence of BOP. In fact, the BOP as a single parameter to define soft‐tissue inflammation might lead to a false‐positive diagnosis [28]. As such, according to the current clinical recommendations, a combination of clinical parameters, including clinical signs of inflammation (i.e., BOP and suppuration) along with the changes in peri‐implant PD values and radiographic marginal bone levels, rather than a single parameter, should be used to monitor peri‐implant tissue conditions [15]. It is worth noting that the outcomes of lateral hard‐tissue augmentation simultaneous with implant placement might be influenced by numerous factors, such as the selection of biomaterials, grafting protocol (i.e., GBR or non‐GBR supported), flap passivation/flap design, defect configuration, and operator‐related factors [4, 7, 9]. Due to the study design, the influence of the dehiscence‐type defect's initial size could not be correlated with peri‐implant tissues' health status. In fact, the defect's morphology was shown to play a crucial role in the regenerative outcome, with advanced and non‐contained defects showing inferior outcomes in terms of defect fill, regardless of the grafting protocol employed [29]. In addition, the present study did not assess the fill of the defects in the LatGr group, which could not allow the association between the extent of the defect fill and peri‐implant tissue health to be evaluated. Finally, the surgical procedures were performed by different operators with various levels of experience, which might have influenced the outcomes obtained, particularly in the LatGr group.

Within the limitations of the present analysis, it can be concluded that lateral hard‐tissue grafting performed simultaneously with implant placement demonstrated comparable peri‐implant tissue health to pristine bone sites without lateral bone grafting.

## Author Contributions

**Amira Begić:** concept, surgeries, patient assessment, data collection, interpretation, and drafting the article. **Frank Schwarz:** concept, data collection, interpretation, drafting the article, and approval of the final version. **Karina Obreja:** patient assessment, data collection, and approval of the final version. **Iulia Dahmer:** statistical analysis, data interpretation, and approval of the final version. **Julie Recktenwald:** data collection, data analysis, and approval of the final version. **Puria Parvini:** data collection, surgeries, data analysis, and approval of the final version. **Ausra Ramanauskaite:** concept, patient assessment, data collection, interpretation, and drafting the article.

## Conflicts of Interest

The authors declare no conflicts of interest.

## Data Availability

The data that support the findings of this study are available from the corresponding author upon reasonable request.
